# Thalamocortical Hyperconnectivity and Amygdala-Cortical Hypoconnectivity in Male Patients With Autism Spectrum Disorder

**DOI:** 10.3389/fpsyt.2019.00252

**Published:** 2019-04-16

**Authors:** Tetsuya Iidaka, Tomohiro Kogata, Yoko Mano, Hidetsugu Komeda

**Affiliations:** ^1^Brain & Mind Research Center, Nagoya University, Nagoya, Japan; ^2^Department of Physical and Occupational Therapy, Graduate School of Medicine, Nagoya University, Nagoya, Japan; ^3^Department of Education, Psychology, and Human Studies, Aoyama Gakuin University, Tokyo, Japan

**Keywords:** resting, functional magnetic resonance imaging, age, development, network, amygdala

## Abstract

**Background:** Analyses of resting-state functional magnetic resonance imaging (rs-fMRI) have been performed to investigate pathophysiological changes in the brains of patients with autism spectrum disorder (ASD) relative to typically developing controls (CTLs). However, the results of these previous studies, which have reported mixed patterns of hypo- and hyperconnectivity, are controversial, likely due to the small sample sizes and limited age range of included participants.

**Methods:** To overcome this issue, we analyzed multisite neuroimaging data from a large sample (n = 626) of male participants aged between 5 and 29 years (mean age = 13 years). The rs-fMRI data were preprocessed using SPM12 and DPARSF software, and signal changes in 90 brain regions were extracted. Multiple linear regression was used to exclude the effect of site differences in connectivity data. Subcortical–cortical connectivity was computed using connectivities in the hippocampus, amygdala, caudate nucleus, putamen, pallidum, and thalamus. Eighty-eight connectivities in each structure were compared between patients with ASD and CTLs using multiple linear regression with group, age, and age × group interactions, head movement parameters, and overall connectivity as variables.

**Results:** After correcting for multiple comparisons, patients in the ASD group exhibited significant increases in connectivity between the thalamus and 19 cortical regions distributed throughout the fronto-parietal lobes, including the temporo-parietal junction and posterior cingulate cortices. In addition, there were significant decreases in connectivity between the amygdala and six cortical regions. The mean effect size of hyperconnectivity (0.25) was greater than that for hypoconnectivity (0.08). No other subcortical structures showed significant group differences. A group-by-age interaction was observed for connectivity between the thalamus and motor-somatosensory areas.

**Conclusions:** These results demonstrate that pathophysiological changes associated with ASD are more likely related to thalamocortical hyperconnectivity than to amygdala-cortical hypoconnectivity. Future studies should examine full sets of clinical and behavioral symptoms in combination with functional connectivity to explore possible biomarkers for ASD.

## Introduction

Autism spectrum disorder (ASD) is characterized by atypical social communication and restricted patterns of behavior, interest, or activities, both of which must be present in the early developmental period (1). Hyper- or hyporeactivity to sensory stimuli and unusual interest in sensory aspects of the environment are highly significant symptoms that are directly associated with subjective distress in daily life in affected patients (2). The distress caused by particular sensory stimuli can cause maladaptive behaviors in those who are unable to communicate appropriately in social situations. Although sensory hyper- and hyporesponsiveness are not unique to ASD, they appear to be more prevalent in this population than among individuals with other developmental disabilities or schizophrenia. Previous studies have investigated the neurophysiological basis of such disturbances in unimodal and multimodal sensory processing among patients with ASD, as well as disturbances in shifting attention to and from sensory stimuli (3, 4).

Extensive structural and functional neuroimaging studies have investigated alterations in patterns of brain connectivity in patients with ASD, relative to typically developing controls. Such studies often employ resting-state functional magnetic resonance imaging (rs-fMRI), a powerful tool for functional connectivity (FC) analysis that may help to elucidate pathophysiological correlates in the brains of patients with ASD. Both hypo- and hyperconnectivity have been observed in brain regions implicated in ASD. That is, the results of each study range from robust underconnectivity to robust overconnectivity depending on the age of participants and type of analysis pipeline. Indeed, previous research has indicated that, in FC analyses, overconnectivity or underconnectivity in patients relative to controls depends on the application of bandpass filtering and task regressors (5). In addition, the use of global signal regression (GSR) has always been debated because the global signal may also include neuronal activity within the whole brain (6). Thus, methodological variables have exerted indispensable effects on group differences reported in previous studies.

Mixed results have also been reported regarding differences in FC between patients with ASD and controls. For example, Glerean et al. suggested that the mixture of hypo- and hyperconnectivity reported across previous ASD studies is reflected in the composition of the default-mode and ventro-temporal-limbic subnetworks (7). In this study, the ASD group exhibited reduced interhemispheric connectivity in regions of typically high interhemispheric connectivity and increased interhemispheric connectivity in areas of typically reduced connectivity (8). In contrast, a study by Tyszka et al. demonstrated that neurotypical and high-functioning adults with autism displayed very similar patterns and strengths of resting-state connectivity, reporting no evidence for altered connectivity at the whole-brain level (9).

These seemingly contradictory findings may be the result of the small sample sizes utilized in previous studies. Abraham et al. demonstrated the high classification accuracy of ASD as compared with controls using a large (n = 871) multisite dataset known as the Autism Brain Imaging Data Exchange (ABIDE) database (10). In another study involving a larger sample size, 418 patients with ASD and 509 matched controls underwent whole-brain voxel-based rs-fMRI, which revealed that patients with ASD exhibited reduced cortical connectivity in the middle temporal gyrus/superior temporal sulcus and increased connectivity in the medial thalamus (11). Such findings indicate that the use of a large multisite dataset may help to elucidate the functional changes in brain connectivity associated with ASD.

A small sample size and limited age range make it difficult to reproduce the studies, as disease-specific patterns of brain activity may change across age groups, and analyses using multiple tests of connectivity fail after correcting for multiple comparisons. To overcome these issues, we analyzed a large rs-fMRI dataset from the ABIDE II database that included only male patients ranging in age from 5 to 29 years. Our primary hypothesis was that neurophysiological changes observed in the brains of patients with ASD are caused by dysconnectivity (hypo- and/or hyperconnectivity) between subcortical structures and cortical mantles. This hypothesis was driven by a recent developmental theory, which proposes that functional imbalances between the affective control system involving subcortical structures (e.g., amygdala and striatum) and the cognitive control system mediated by the prefrontal cortices may affect emotional and social behaviors during early to late adolescence (12). According to this model, sensory modulation in ASD (2, 3) most likely involves functional alterations in thalamocortical connectivity, as the thalamus is known to connect primary sensory input with higher-order cortical areas (13–15).

## Materials and Methods

### Participants

The original imaging and demographic data were collected from the ABIDE II database (http://fcon_1000.projects.nitrc.org/indi/abide/index.html), which allows unrestricted usage for noncommercial research purposes. Although the dataset included both adults and children of both sexes, only male participants ranging from 5 to 29 years of age were included in the present study. Brain images and related data from 368 patients with ASD and 362 control participants with typical development (CTL) from 17 universities and research institutes were used for the initial analysis. Following exclusion of participants with excessive head movement during scanning and failure in the spatial normalization steps (for details, see the section Imaging Data Analysis), the results from the remaining 311 patients with ASD and 315 CTLs were reported. The mean ages ( ± s.d). in the ASD and CTL groups were 13.9 ± 5.3 and 13.4 ± 5.5 years, respectively. The participants’ demographic data and the abbreviated names of each institution are listed in **Supplementary Table 1**. The ethics committee of the Nagoya University School of Medicine approved the usage of these anonymous data for research purposes.

Autism was diagnosed using the Autism Diagnostic Interview-Revised (ADI-R) (16) and Autism Diagnostic Observation Schedule (ADOS) (17) in almost all cases. The CTL participants were screened in clinical interviews conducted by experts in child psychiatry. Intelligent quotients (IQs) were measured in 301 patients with ASD and 310 CTLs; however, all but one institute provided full scale IQ, while one institute (EMC) provided only performance IQ. Details regarding the diagnostic procedures and questionnaires used can be found on the ABIDE website. Age and IQ distribution for each group are shown in **Supplementary Figure 1**. There was no significant difference in mean age between the two groups (t-test, *p* = 0.24), although IQ was lower in the ASD group than in the CTL group (mean ± s.d. ASD; 106 ± 16, CTL; 114 ± 12, t-test, *p* < 0.001). Among the 311 patients with ASD, the ADOS score (17) was obtained from 188 (60%) patients. Ninety-four patients with ASD (30%) were receiving psychotropic medication at the time of scanning, while 185 (60%) were not. Data for medication status were unavailable for 32 (10%) patients. Among CTL participants, 12 (4%) were taking medication at the time of scanning, while 260 (83%) were not. Data for medication status were unavailable for 43 (14%) CTLs (**Supplementary Figure 2**).

### Imaging Data Acquisition

At each institute, functional brain images were acquired using a 3-T scanner and a T2*-weighted gradient-echo echo-planar imaging (EPI) sequence, which is sensitive to blood oxygen level-dependent (BOLD) contrast. Participants were asked to lie still in the scanner while remaining awake. Although the scanning parameters, MRI vendor, voxel size, number of volumes, scanning time, and instructions whether to keep eyes open/closed varied among the institutes, the general experimental procedure used was uniform within each institute. The number of image volumes for each participant ranged from 120 to 947 (mean = 234), and scanning time ranged from 5 to 16.4 min (mean = 7.0). The details of the scanning parameters and experimental settings are provided in **Supplementary Table 2**.

### Imaging Data Analysis

#### Preprocessing

Data were analyzed using SPM12 software (Wellcome Department of Imaging Neuroscience, London, UK, http://www.fil.ion.ucl.ac.uk/spm/) at the Brain and Mind Research Center of Nagoya University. After discarding the first 10 volumes, all volumes were spatially realigned to the mean volume, and the signal in each slice was temporally realigned to that obtained in the middle slice using sinc interpolation. No slice timing correction was applied for datasets with multiband acquisition. The resliced volumes were normalized to the Montreal Neurological Institute (MNI) space with a voxel size of 3 × 3 × 3 mm using an EPI template in SPM12. The normalized images were spatially smoothed with a 4-mm Gaussian kernel. After the preprocessing steps, several quality control steps were employed. Participants whose maximum head movement was greater than 3.0 mm and 3° and those whose normalized images did not correctly match the EPI template were removed from the study.

### Resting-State Functional Connectivity Analyses

Preprocessed datasets were further processed using the Data Processing Assistant for Resting-State fMRI toolkit (DPARSF; http://www.rfmri.org, advanced version) (18). Processing was conducted according to the following steps: 1) removal of the linear and quadric trends in the time series; 2) temporal band-pass filtering (0.01–0.1 Hz) to reduce the effect of low-frequency drift and high-frequency noise; 3) regressing out the effect of head motion during scanning using six head motion parameters, six head motion parameters one time point before, and the 12 corresponding squared items (Friston 24-parameter model); and 4) controlling for nonneural noise in the time series by including covariates in the linear regression (i.e., the white matter and cerebrospinal fluid signals). Since sufficient empirical evidence has shown that the results of FC after GSR should be interpreted carefully, the global signal was not removed in the present study (6, 19, 20). Head motion is known to have substantial effects on the results of FC analyses (21). Image scrubbing of the time-series data was performed by deleting one time point before and two time points after the excessive head movement, as defined by framewise displacement (FD) greater than 0.5 (22). The group differences in mean FD value and the proportion of scrubbed volumes were investigated using a Mann–Whitney U-test (statistical threshold, *p* < 0.05, **Table 1**).

**Table 1:** Group differences in head motion parameters.

	ASD	CTL	U-test
FD	0.22 (0.20)	0.19 (0.11)	*p* = 0.051
Scrubbed (%)	18.3 (17.8)	14.9 (16.7)	*p* = 0.002

FD, frame wise displacement; ASD, autism spectrum disorder; CTL, control. Scrubbed (%): the proportion of the scrubbed volumes in total scan volumes. Mean and SD in the parentheses. U-test: Mann–Whitney U-test.

The residuals of the datasets after band-pass filtering, removal of the trends and nuisance covariates, and scrubbing were regarded as BOLD signal fluctuations originating from neuronal activity during the resting state. The Automated Anatomical Labeling (AAL) template (23), which is widely used for identifying brain regions in the MNI space, was applied to the normalized and smoothed time-series datasets of each participant. The AAL template is a standard brain template for creating intrinsic connectivity (24, 25), although other templates have been used for the same purpose [e.g., the Harvard-Oxford Atlas (9, 26) and voxel-wise lattice method (27, 28)]. The AAL template divides each hemisphere into 45 distinct regions. The average time-series data were computed in each of the 90 regions for each participant. We used the AAL template because it is implemented in DPARSF software, and a 90 × 90 correlation matrix is better suited to avoid issues with multiple comparisons in the data analysis, relative to other templates with higher spatial resolution.

The time-series data from each of the 90 regions were cross-correlated, and Pearson’s correlation coefficients (*r*) between each brain region and the remaining 89 regions were computed for each participant. Individual *r* values were normalized to *z* values using Fisher’s *z*-transformation. The *z*-transformed correlation coefficients were represented in a 90 × 90 FC matrix, which was symmetric with regard to the diagonal. Multiple linear regression was used to further control for site effect on the FC matrix by regressing out the *z*-transformed correlation coefficients for each matrix element on dummy variables for each site. The standardized residuals were used as an estimate of the FC of each region and every other region controlling for site effect (29). This process was conducted using MATLAB-based in-house software.

### Extracting Subcortical–Cortical Functional Connectivity

Our *a priori* hypothesis was that subcortical–cortical connectivity is altered in patients with ASD relative to CTLs during the developmental period and early adulthood. Therefore, we extracted the subcortical–cortical connectivity values from the matrix elements created in the previous section. Six subcortical structures (i.e., the hippocampus, amygdala, caudate nucleus, putamen, pallidum, and thalamus) in the left and right hemispheres, as defined by the AAL template, were selected for this purpose. For each structure, 88 FC values with every other region were extracted by excluding the FC with a counterpart in an opposite hemisphere; for example, an FC between the left and right hippocampus was not included in the analysis. Finally, the FCs from subcortical structures in each of the left and right hemispheres were averaged; for example, we averaged connectivity values between the left hippocampus and region A and those between the right hippocampus and region A. Finally, overall mean connectivity, i.e., the average of all elements of the 6×88 subcortical and cortical connectivity matrix, was computed in each subject.

### Group Differences in Subcortical–Cortical Functional Connectivity

**Figure 1:**
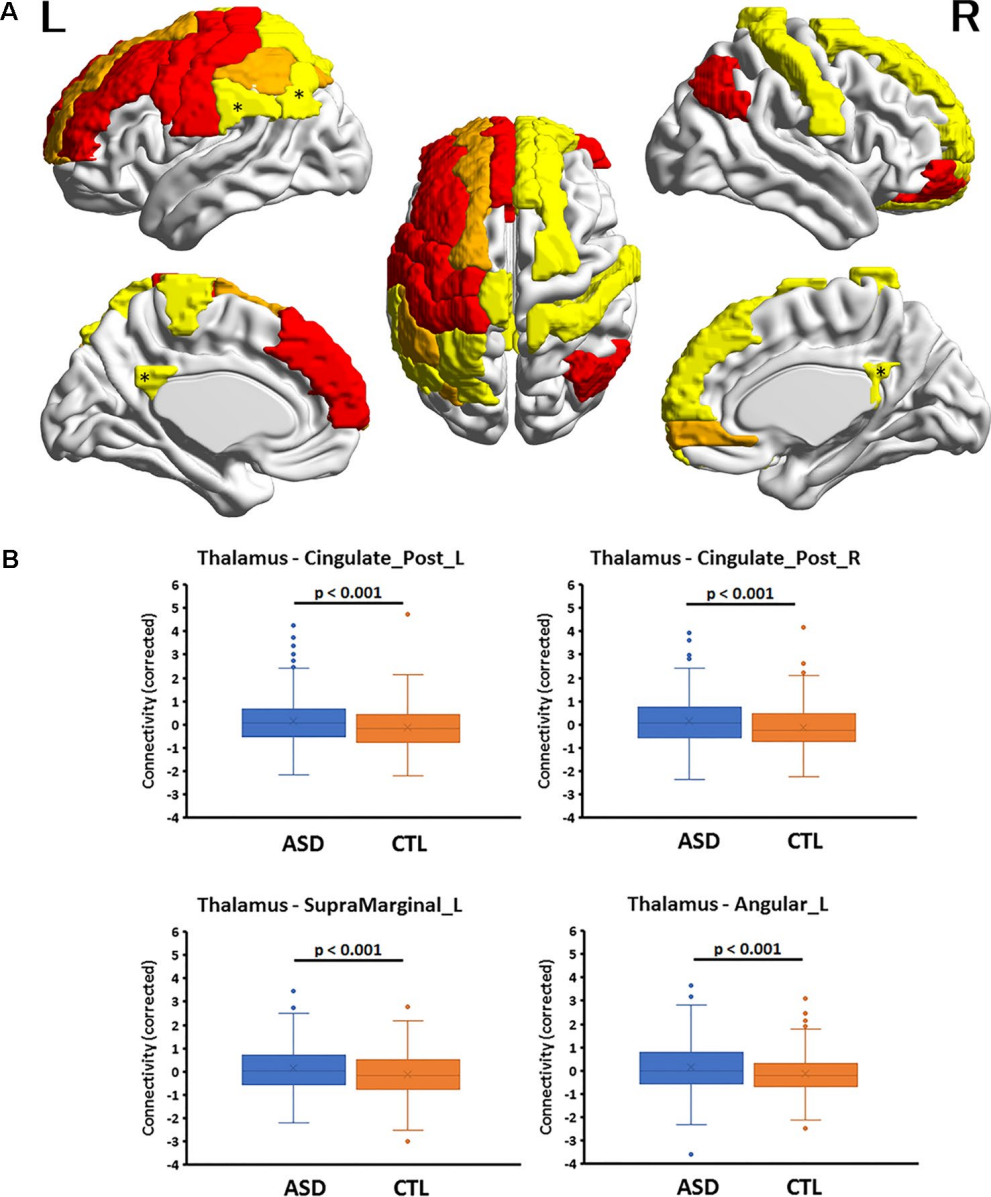
**(A)** Cortical regions with hyperconnectivity with the thalamus in autism. Nineteen cortical regions in which connectivity with the thalamus was significantly greater for the autism spectrum disorder (ASD) group than for the control (CTL) group after false-discovery rate (FDR) correction were mapped on the surface of the template brain. Colored regions correspond to the regions listed in **Table 1**. The colors of the regions indicate levels of FDR adjusted *p*-value—yellow: 0.02 < *p* < 0.03; orange: 0.03 < *p* < 0.05; red: 0.04 < *p* < 0.05. An asterisk indicates the region shown in the bottom. The figure was created using BrainNet Viewer. **(B)** Four representative regions with thalamocortical hyperconnectivity in autism. Boxplots of four thalamocortical functional connectivities for the ASD and CTL groups. Among the 19 connectivities that survived FDR correction, these 4 were highly significant (Hedges’s *g* > 0.27) and were associated with the posterior cingulate cortex and temporo-parietal junction (TPJ, supramarginal and angular gyri), which are subdivisions of the default mode network. The mean connectivity values were significantly higher in the ASD group than in the CTL group. A boxplot shows the distribution of data into quartiles, the mean (cross;×), and outliers. The whisker lines indicate the outer boundaries of the upper and lower quartiles, while points outside the whiskers indicate outliers. These are representative results, and all connectivities that survived FDR correction are listed in **Table 1**.

**Figure 2:**
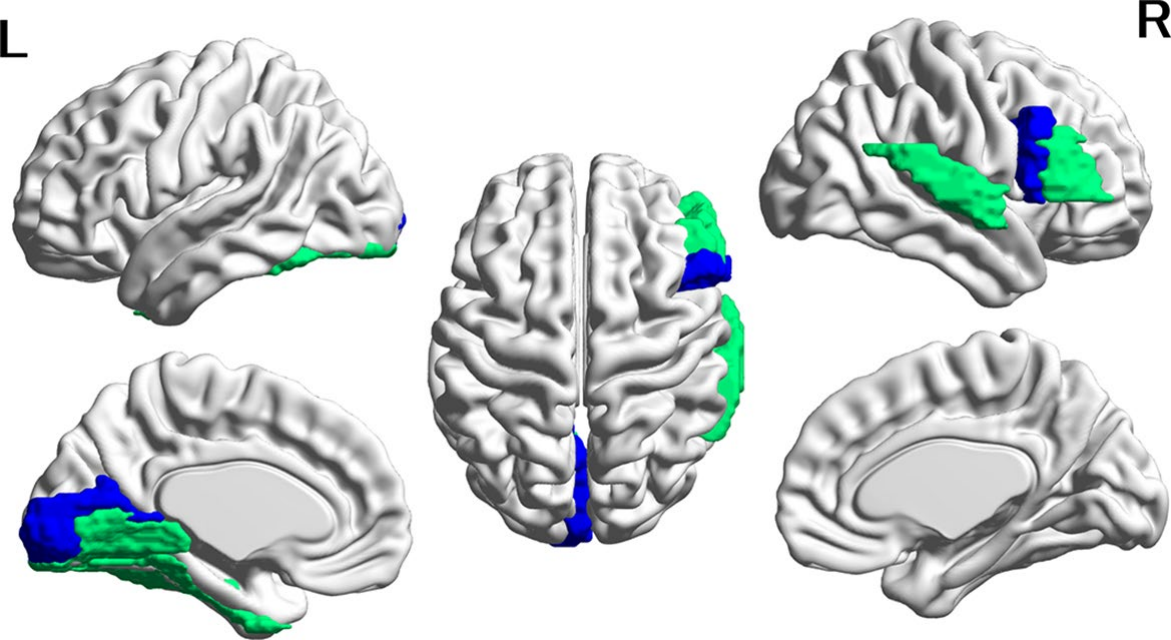
Cortical regions with hypoconnectivity with the amygdala in autism. Six cortical regions in which connectivity with the amygdala was significantly greater for the CTL group than for the ASD group after FDR correction were mapped on the surface of the template brain. Colored regions correspond to the regions listed in **Table 2**. The colors of the regions indicate levels of FDR adjusted *p*-value—green: 0.02 < *p* < 0.03; blue: 0.03 < *p* < 0.04. The figure was created using BrainNet Viewer.

Eighty-eight FCs in each of the six structures were compared between the ASD and CTL groups *via* multiple linear regression using the FC as a dependent variable and group, age, the age × group interaction, mean FD, and overall mean connectivity as independent variables. Age, mean FD, and overall mean connectivity were mean corrected. The analysis was conducted using Origin Pro software (https://www.originlab.com/). The statistical threshold was set at *p* < 0.05 after correction for multiple comparisons using a false-discovery rate (FDR) (30) in each structure. In the present study, a significant main effect of group on FC strength was obtained only for the thalamus and amygdala. The region names, adjusted *p*-values of the main effect of group, and the effect size of Hedges’s *g* are listed in **Tables 2** and **3**. The cortical regions that survived the FDR correction were superimposed on the cortical surface using BrainNet Viewer (**Figures 1** and **2**, http://www.nitrc.org/projects/bnv/) (31). Among the significant results for the thalamus, FC values for four cortical regions with an effect size *g* > 0.27 are plotted in **Figure 1**. To investigate whether the significant results could be influenced by the group difference in intellectual function, FIQ was added as an independent variable in the multiple regression analysis. Finally, Pearson’s correlation coefficients between the significant thalamocortical and amygdala-cortical connectivity values and social and communication subscales of the ADOS were investigated (statistical threshold, *p* < 0.05).

**Table 2:** Cortical regions with significant hyperconnectivity with thalamus in ASD group.

No.	Region name	adj-p-value	E.S. (*g*)
1	Precentral_L	0.048	0.21
2	Frontal_Sup_L	0.033	0.23
3	Frontal_Sup_R	0.028	0.25
4	Frontal_Sup_Orb_R	0.025	0.25
5	Frontal_Mid_L	0.040	0.23
6	Frontal_Mid_Orb_R	0.042	0.24
7	Frontal_Sup_Medial_L	0.049	0.22
8	Frontal_Sup_Medial_R	0.023	0.26
9	Frontal_Med_Orb_R	0.033	0.22
10	Cingulum_Post_L	0.025	0.27
11	Cingulum_Post_R	0.023	0.28
12	Postcentral_L	0.043	0.24
13	Postcentral_R	0.023	0.26
14	Parietal_Sup_L	0.025	0.24
15	Parietal_Inf_L	0.033	0.24
16	SupraMarginal_L	0.023	0.29
17	Angular_L	0.023	0.27
18	Angular_R	0.049	0.23
19	Paracentral_Lobule_L	0.028	0.25

All p-values are adjusted and significant after FDR correction at p < 0.05. E.S. (g) indicates effect size of Hedges’s g. Positive effect size indicates hyperconnectivity in ASD and negative effect size indicates hypoconnectivity in ASD as compared with CTL.

**Table 3:** Cortical regions with significant hypoconnectivity with amygdala in ASD group.

No.	Region name	adj-*p*-value	E.S. (*g*)
1	Frontal_Inf_Oper_R	0.039	−0.07
2	Frontal_Inf_Tri_R	0.028	−0.07
3	Calcarine_L	0.039	−0.07
4	Lingual_L	0.020	−0.10
5	Fusiform_L	0.023	−0.09
6	Temporal_Sup_R	0.020	−0.08

All p-values are adjusted and significant after FDR correction at p < 0.05. E.S. (g) indicates effect size of Hedges’s g. Positive effect size indicates hyperconnectivity in ASD and negative effect size indicates hypoconnectivity in ASD as compared with CTL.

### Effect of Age and Age-by-Group Interaction on Functional Connectivity

**Figure 3:**
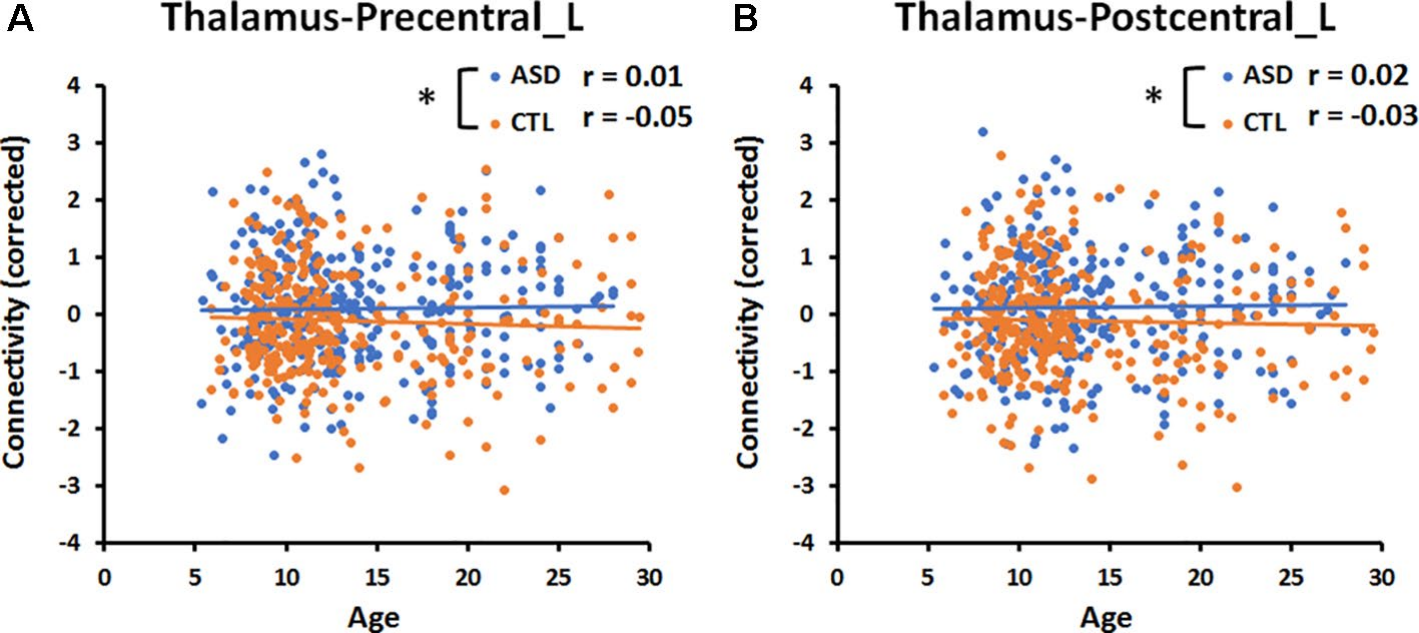
Significant age-by-group interaction effect on thalamocortical connectivities. **(A)** The correlation between age and connectivity in the thalamus-precentral gyrus of the left hemisphere was weakly negative (*r* = −0.05) in the CTL group, but near zero (*r* = 0.01) in the ASD group. **(B)** The correlation between age and thalamus-postcentral gyrus of the left hemisphere was weakly negative (*r* = −0.03) in the CTL group, but weakly positive (*r* = 0.02) in the ASD group. An asterisk indicates a significant age-by-group interaction effect (*p* < 0.05, uncorrected). Blue and orange lines indicate the regression lines of ASD and CTL, respectively.

Multiple linear regression using the FC values from the thalamus and amygdala revealed significant group differences in FC following FDR correction for multiple comparisons. Because these results included a main effect of age and age-by-group interaction terms, we used the main effect and interaction to investigate the region in which the relationship between connectivity and age may or may not differ between the ASD and CTL groups. The cortical regions in which the main effect and interaction terms were significant in the multiple linear regression analysis were reported. The statistical threshold was set at *p* < 0.05, uncorrected for multiple comparisons. For the significant age-by-group interaction effect, the FC values and age for both groups are plotted in **Figure 3**. Pearson’s correlation coefficient between age and FC in each group was computed without testing the statistical significance level because the interaction term was proven to be significant in the multiple regression analysis.

### The Relationship among Age, Head Movement, Intellectual Function, and Connectivity

To explore the relationships among the age, FD, FIQ, and overall connectivity, Pearson’s correlation coefficients were computed for each group (statistical threshold, *p* < 0.05, **Supplementary Table 4**). A multiple regression analysis was conducted including each of these values (age, FD, FIQ, and overall connectivity) as a dependent variable and age, group, and the age × group interaction term as independent variables (statistical threshold, *p* = 0.05).

### Effects of Medication on Functional Connectivity in the ASD Group

In the present study, we included participants receiving any kind of psychotropic medication that may alter the FC in the brain because the exclusion of those participants would have reduced the sample size by 29%, potentially decreasing the statistical power of the dataset. To clarify the effects of medication on our results, we compared FC matrices between 94 patients with ASD who received psychotropic medication (on-med group) and 185 who did not (off-med group) at the time of scanning. The mean age and mean IQ were compared between the groups using t-tests. Eighty-eight FCs in the thalamus and amygdala were compared between the on- and off-med groups using the FC as a dependent variable, and group, age, the age × group interaction, mean FD, and overall mean connectivity as independent variables. Age, mean FD, and overall mean connectivity were mean corrected. Finally, mean FD and the proportion of scrubbed volumes were compared between the on- and off-med groups using a Mann–Whitney U-test (**Supplementary Table 5**). The statistical threshold was set at *p* < 0.05, uncorrected.

## Results

### Group Differences in Head Motion Parameters

There was a trend towards significance (*p* = 0.051) in the difference in FD values between the ASD and CTL groups, and the proportion of scrubbed volumes was significantly higher in the ASD group than in the CTL group (**Table 1**).

### Group Differences in Subcortical–Cortical Functional Connectivity

Multiple linear regression analyses revealed that, among the six subcortical structures including the amygdala, hippocampus, caudate nucleus, pallidum, putamen, and thalamus, only those FCs connecting the thalamus and amygdala with several cortical regions exhibited significant main effects of group after correcting for multiple comparisons. In all 19 thalamocortical connectivities that survived correction for multiple comparisons, the mean FC values were greater in the ASD group than in the CTL group. The effect size of the group difference ranged from 0.21 to 0.29 (mean, 0.25, **Table 2** and **Figure 1**). In the ASD group, increased connectivity was observed between the thalamus and the frontal, cingulate, and parietal cortices of the left and right hemispheres. Regions exhibiting highly significant differences and medium effect sizes were identified in the left and right posterior cingulate cortices (PCCs; Cingulum_Post_L and Cingulum_Post_R), left supramarginal gyrus (SupraMarginal_L), and left angular gyrus (Angular_L). Boxplots of the FC values in the four regions for both groups are illustrated in **Figure 1**.

In contrast, in all six amygdala-cortical connectivities that survived correction for multiple comparisons, the mean FC values were lower in the ASD group than in the CTL group. The effect size of the group difference ranged from 0.07 to 0.10 (mean, 0.08, **Table 3** and **Figure 2**). In the ASD group, decreased connectivity was observed between the amygdala and the frontal, occipital, and temporal cortices of the left and right hemispheres. When the FIQ was included as an independent variable in the multiple regression analysis of these connectivities, the group difference remained significant (uncorrected *p*-values: 0.0007–0.025). There was no significant correlation between the connectivity values and the ADOS subscales (social and communication). The results of group difference in connectivity that did not survive FDR correction at *p* < 0.05 but were significant at uncorrected *p* < 0.05 are listed in **Supplementary Table 3**.

### Effect of Age and Age-by-Group Interaction on Functional Connectivity

The multiple linear regression analysis revealed a significant (*p* < 0.05, uncorrected) age-by-group interaction for the connectivity between the thalamus and left precentral gyrus (Precentral_L, *p* = 0.02, **Figure 3**), and for that between the thalamus and left postcentral gyrus (Postcentral _L, *p* = 0.02, **Figure 3**). The correlation between age and the connectivity between the thalamus and left pre- and postcentral gyri was near zero in the ASD group and weakly negative in the CTL group. There was no significant age-by-group interaction for the connectivity between the amygdala and six cortical regions.

The multiple linear regression analysis revealed a significant (*p* < 0.05, uncorrected) main effect of age for the connectivity between the thalamus and right superior frontal gyrus (Frontal_Sup_R, *r* = 0.04, *p* = 0.01), for that between the thalamus and right orbital gyrus (Frontal_Mid_Orb_R, *r* = 0.06, *p* = 0.04), and for that between the thalamus and left paracentral lobule (Paracentral_Lobule_L, *r* = 0.05, *p* = 0.02). There was no significant main effect of age for the connectivity between the amygdala and six cortical regions.

### The Relationship among Age, Head Movement, Intellectual Function, and Connectivity

In the ASD group, there was a significant negative correlation between FD and age (*r* = −0.17, *p* = 0.003) and between FD and mean connectivity (*r* = 0.27, *p* < 0.001). In the CTL group, there was a significant negative correlation between the FD and FIQ (*r* = −0.13, *p* = 0.02) and between FD and mean connectivity (*r* = 0.26, *p* < 0.001). However, there was no significant group-by-age interaction effect on these correlations. For other insignificant results, see **Supplementary Table 4**.

### Effects of Medication on Functional Connectivity in the ASD Group

No significant difference in mean age was observed between the on-med and off-med ASD groups (on-med, 13.2 ± 4.1, off-med, 13.5 ± 5.4, *p* = 0.65, **Supplementary Figure 3**, left); however, mean IQ significantly differed between the groups (on-med, 109 ± 17, off-med, 105 ± 16, *p* = 0.045, **Supplementary Figure 3**, right). Mean FD and the proportion of scrubbed volumes did not significantly differ between the on- and off-med groups (**Supplementary Table 5**). The multiple linear regression analysis revealed no significant main effect of medication on the FCs between the thalamus/amygdala and cortical regions (all *p *> 0.05, uncorrected). These results suggest that the effect of psychotropic medication on thalamocortical connectivity was relatively negligible in the present study.

## Discussion

In the present study, we analyzed a large rs-fMRI dataset from the ABIDE II database to determine whether neurophysiological changes observed in the brains of patients with ASD are caused by dysconnectivity between subcortical and cortical structures. Our findings indicated that the ASD group exhibited significantly increased connectivity between the thalamus and 19 cortical regions after FDR correction for multiple comparisons, relative to that observed in the CTL group. The mean effect size of the group difference was 0.25, and, therefore, the degree of difference was not small or negligible. The cortical regions comprised the frontal and parietal areas as well as the supramarginal and angular gyrus (i.e., temporo-parietal junction, TPJ) and bilateral PCCs. In the pre- and postcentral gyri of the left hemisphere, there were significant group differences in the relationship between connectivity and the participant’s age, indicating that connectivity decreases as age increases among typically developing CTLs, but not among patients with ASD.

In addition, the present study revealed that the ASD group exhibited significantly decreased connectivity between the amygdala and six cortical regions after FDR correction for multiple comparisons, relative to that observed in the CTL group. However, the mean effect size of the group difference was 0.08, and, therefore, the degree of difference in amygdala-cortical connectivity is smaller than that for thalamocortical connectivity. These group differences in connectivity were not influenced by the difference in intellectual function between the groups.

Thus, the results of the present study suggest that hyperconnectivity in thalamocortical pathways, and to a lesser extent hypoconnectivity in amygdala-cortical pathways, are associated with the pathophysiology of ASD. Furthermore, as the present results were obtained after regressing out the mean FD and overall mean connectivity values, the group differences in connectivity should be unrelated to the differences in head movement and regionally specific to the thalamus and amygdala.

Previous studies have reported conflicting results regarding patterns of hypo- and hyperconnectivity in patients with ASD. Most of these studies have observed reduced functional and structural connectivity among patients with ASD (32–35), while relatively few have described hyperconnectivity (36–38). However, such studies may not have sufficient statistical power due to the small sample size and limited age range, necessitating confirmation regarding the reliability and reproducibility of the findings. A very recent rs-fMRI study demonstrated that the FC between the thalamus and the primary and secondary auditory cortices was significantly higher in the ASD group than in the CTL group (39). Therefore, the results of the present study indicate that widespread cortical areas are overly connected to the thalamus, further supporting the notion of thalamocortical hyperconnectivity among patients with ASD.

Several other large-scale studies have used multisite neuroimaging data to investigate the neural underpinnings of ASD (11, 40, 41). Cheng et al. investigated differences in FC between 418 patients with ASD and 509 CTLs (11), reporting both hyper- and hypoconnectivity in the ASD group. However, the authors noted that the thalamus exhibited significantly greater connectivity with the rest of the brain in the ASD group than in the CTL group. Although our findings regarding thalamic hyperconnectivity were similar to those of Cheng et al. (11), they were unable to elucidate which regions of the brain were hyperconnected to the thalamus because their connectivity value was the average of the whole brain. In a study by Woodward et al., an older version of the ABIDE I database was used for analysis (228 patient–control pairs). The authors investigated only the cortical connectivity from and to the thalamus and revealed, similar to the present study, increased prefrontal, motor, somatosensory, and temporal cortex connectivity with the thalamus in the autism group as compared with the control group (41).

In a study by Cerliani et al. involving 166 patients with ASD and 193 CTLs (all male, age range: 6 to 50 years), the authors performed an independent component analysis (ICA) using an ABIDE dataset (40). One component involving the thalamus and basal ganglia exhibited significantly greater connectivity with several other components involving the motor, visual, and auditory areas as well as the superior temporal sulcus in the ASD group than in the CTL group. Their results are relevant to the results of the present study; however, the critical component did not differentiate the thalamus and other basal ganglia regions when the ICA was applied to the rs-fMRI data. In another recent study, Fu et al. observed dynamic hyperconnectivity between the hypothalamus/subthalamic region and several sensory areas (42). Taken together, the results of studies utilizing large multisite datasets indicate that ASD is associated with thalamocortical hyperconnectivity, rather than hypoconnectivity.

Thalamocortical pathways are critical to sensory processing and perception because they carry information received from the primary sensory organs to cortical areas specific to each sensory modality. Thalamic input to the cortex is important for the development of sensory and association cortices, and the thalamus appears to influence the formation of sensory representation in the cortices during early development (15). Therefore, abnormalities in sensory processing, which have been widely associated with ASD (4, 39), may be caused by developmental irregularities within the neuronal connectivity between the thalamus and sensory areas in the cortex. Additional studies have indicated that the thalamic reticular nucleus (TRN), which surrounds the thalamic projection circuit with inhibitory GABAergic neurons, may play a critical role in attention and sensory processing (14). The disruption of inhibitory mechanisms involving the TRN in patients with ASD may give rise to sensory hyperarousal and the hyperconnectivity patterns observed using rs-fMRI.

The mediodorsal nucleus (MDN) of the thalamus plays a key role in higher-order cognitive functions, such as working memory and attention, mechanisms of which appear to be based on the relationship between the MDN and the frontal and cingulate cortices. The MDN may interact with prefrontal cortices during the performance of higher-order cognitive tasks, and is also likely involved during social communication. The researchers have proposed that disruptions in cortico-cortical connectivity may occur during the early neurodevelopmental period, and that such disruptions are caused by alterations in connectivity between the thalamus and the cortex. Such significant alterations in cortico-cortical communication may contribute to many of the cognitive disruptions evident in neurodevelopmental disorders (43).

In the present study, the most significant differences between the ASD and CTL groups were observed in the TPJ and the posterior cingulate cortex. These areas are subcomponents of the default mode network and are considered to be related to several specific mental processes such as self-related thoughts and mind-wandering (44). Specifically, the TPJ is implicated in a variety of processes including multisensory integration and social cognition, most of which are impaired in patients with ASD (45). These findings indicate that the heightened connectivity observed in the ASD group of the present study may reflect TPJ malfunctioning.

On the other hand, a study that examined the FC patterns of the amygdala using rs-fMRI demonstrated that adolescents with ASD have decreased FC between the amygdala and the thalamus/putamen compared to CTLs (46). The patients with ASD showed weaker fronto-amygdala connectivity in the right hemisphere (47). There was a significant reduction in connectivity between the amygdala and prefrontal, parietal, and occipital cortices in the ASD group compared with the CTL group (48, 49). In a study using younger subjects (mean age: 3.5 years), the ASD group showed significantly weaker connectivity between the amygdala and medial prefrontal cortices (50). The results of previous studies are in accordance with those of the present study showing a reduced connectivity pattern between the amygdala and several cortical regions in a large sample of patients with ASD. Although the amygdala is a critical structure involved in emotional perception and regulation in patients with ASD (47–50), the small effect size observed in the present study may preclude the possibility that the amygdala plays a principal role in the pathogenesis of the disease.

Notably, we observed a significant group-by-age interaction effect on patterns of FC in the pre- and postcentral gyri. These interaction effects are most likely derived from decreases in connectivity with age in the CTL group and fixed connectivity in the ASD group. Our findings suggest that this age range represents a critical period for neuronal development and maturation in CTLs. Previous reviews of age-related differences in brain structure have reported that gray matter volume decreases, while white matter volume increases, during puberty and early adolescence (51, 52). Therefore, unchanging connectivity may reflect neurodevelopmental delays or irregularities observed in the ASD group in this study.

The present study possesses several limitations of note. First, in order to maintain the statistical power of the large multisite dataset, we did not exclude patients taking medication from the study population. However, we observed no statistically significant differences in thalamocortical or amygdala-cortical connectivity between the medication groups, suggesting that medication exerts virtually no influence on connectivity in patients with ASD. Second, there was no significant correlation between the connectivity values and clinical symptoms in the ASD group. While we could not reveal the neural substrates of such symptoms in the ABIDE II database, future datasets that provide complete sets of symptom scales may enable us to reveal the relationship between connectivity and symptoms. Third, in the present study, the brain template did not differentiate subnuclei within the thalamus. Therefore, the resultant activity was an average of that in the whole thalamus. This may obscure the exact pattern of FC, as the thalamus is composed of several nuclei, each of which exhibits specific neuronal connections and functions (15, 43). Further analysis using an anatomical template delineating separate thalamic subnuclei (53) is therefore required.

## Conclusions

Using a large dataset from multiple ABIDE II database sites, we demonstrated that hyperconnectivity between the thalamus and fronto-parietal cortices, most notably the left TPJ and bilateral PCC, is associated with the pathophysiology of ASD. Moreover, amygdala-cortical connectivity was significantly decreased in the ASD group. Future studies should collect full sets of clinical and behavioral data in combination with FC data to explore possible biomarkers for ASD.

## Author Contributions

TI contributed to data collection, analyzed and interpreted the data, and wrote the manuscript. TK contributed to the methods and data preparation. YM and HK made substantial contributions to interpretation of data and wrote the manuscript. All authors were involved in drafting the manuscript, and read and approved the final manuscript.

## Funding

This study was supported by KAKENHI No. 17H05923 and No. 15H01846.

## Conflict of Interest Statement

The authors declare that the research was conducted in the absence of any commercial or financial relationships that could be constructed as a potential conflict of interest.

## Supplementary Material

Supplementary Table 1 Participants demographics.

Supplementary Table 2 Names of institutes and data acquisition parameters.

Supplementary Table 3 Subcortical connectivity which did not survive FDR correction.

Supplementary Table 4 Correlation between FD, age, full-IQ, and mean connectivity.

Supplementary Table 5 Medication effect on head motion parameters.

Supplementary Figure 1 Age and IQ distribution of the participants. Distributions of age at the time of scanning (top) and IQ (bottom) for the ASD (blue column) and CTL groups (orange column). The vertical axis indicates the number of participants. There was no significant group difference in mean age, although mean IQ was significantly lower (*p*< 0.001) in the ASD group than in the CTL group. IQ: intelligence quotient; ASD: autism spectrum disorder; CTL: control.

Supplementary Figure 2 Medication status of participants in each group. The number in the column indicates the number of participants, while the scale below indicates the percentage.

Supplementary Figure 3 Medication effect on age and IQ. Boxplot of age (left) and IQ (right) in the on- and off-medication ASD groups. Patients in the on-med group exhibited significantly higher mean IQ values than those of the off-med group (*p*< 0.05). IQ: intelligence quotient.

## References

[1] American_Psychiatric_Association Diagnostic and statistical manual for mental disorders. 5th edition American Psychiatric Association, Arlington, VA, (2013).

[2] Ben-Sasson A Hen L Fluss R Cermak SA Engel-Yeger B Gal E A meta-analysis of sensory modulation symptoms in individuals with autism spectrum disorders. J Autism Dev Disord (2009) 39:1–11. 10.1007/s10803-008-0593-3 18512135

[3] Marco EJ Hinkley LB Hill SS Nagarajan SS Sensory processing in autism: a review of neurophysiologic findings. Pediatr Res (2011) 69:48R–54R. 10.1203/PDR.0b013e3182130c54 PMC3086654 21289533

[4] Mikkelsen M Wodka EL Mostofsky SH Puts NAJ Autism spectrum disorder in the scope of tactile processing. Dev Cogn Neurosci (2018) 29:140–50. 10.1016/j.dcn.2016.12.005 PMC5481487 28089657

[5] Nair A Keown CL Datko M Shih P Keehn B Muller RA Impact of methodological variables on functional connectivity findings in autism spectrum disorders. Hum Brain Mapp (2014) 35:4035–48. 10.1002/hbm.22456 PMC5708536 24452854

[6] Caballero-Gaudes C Reynolds RC Methods for cleaning the BOLD fMRI signal. Neuroimage (2017) 154:128–49. 10.1016/j.neuroimage.2016.12.018 PMC5466511 27956209

[7] Glerean E Pan RK Salmi J Kujala R Lahnakoski JM Roine U Reorganization of functionally connected brain subnetworks in high-functioning autism. Hum Brain Mapp (2016) 37:1066–79. 10.1002/hbm.23084 PMC6867362 26686668

[8] Hahamy A Behrmann M Malach R The idiosyncratic brain: distortion of spontaneous connectivity patterns in autism spectrum disorder. Nat Neurosci (2015) 18:302–9. 10.1038/nn.3919 25599222

[9] Tyszka JM Kennedy DP Paul LK Adolphs R Largely typical patterns of resting-state functional connectivity in high-functioning adults with autism. Cereb Cortex (2014) 24:1894–905. 10.1093/cercor/bht040 PMC4051895 23425893

[10] Abraham A Milham MP Di Martino A Craddock RC Samaras D Thirion B Deriving reproducible biomarkers from multi-site resting-state data: an Autism-based example. Neuroimage (2017) 147:736–45. 10.1016/j.neuroimage.2016.10.045 27865923

[11] Cheng W Rolls ET Gu H Zhang J Feng J Autism: reduced connectivity between cortical areas involved in face expression, theory of mind, and the sense of self. Brain (2015) 138:1382–93. 10.1093/brain/awv051 PMC4407191 25795704

[12] Shulman EP Smith AR Silva K Icenogle G Duell N Chein J The dual systems model: review, reappraisal, and reaffirmation. Dev Cogn Neurosci (2016) 17:103–17. 10.1016/j.dcn.2015.12.010 PMC6990093 26774291

[13] Ferrarelli F Tononi G The thalamic reticular nucleus and schizophrenia. Schizophr Bull (2011) 37:306–15. 10.1093/schbul/sbq142 PMC3044616 21131368

[14] Krol A Wimmer RD Halassa MM Feng G Thalamic reticular dysfunction as a circuit endophenotype in neurodevelopmental disorders. Neuron (2018) 98:282–95. 10.1016/j.neuron.2018.03.021 PMC6886707 29673480

[15] Lopez-Bendito G Development of the thalamocortical interactions: past, present and future. Neuroscience (2018) 385:67–74. 10.1016/j.neuroscience.2018.06.020 29932982 PMC7611009

[16] Lord C Rutter M Le Couteur A Autism Diagnostic Interview-Revised: a revised version of a diagnostic interview for caregivers of individuals with possible pervasive developmental disorders. J Autism Dev Disord (1994) 24:659–85. 10.1007/BF02172145 7814313

[17] Lord C Rutter M DiLavore PC Risi S Autism diagnostic observation schedule. Los Angeles: Western Psychological Service (1999).

[18] Chao-Gan Y Yu-Feng Z DPARSF: A MATLAB Toolbox for “pipeline” data analysis of resting-state fMRI. Front Syst Neurosci (2010) 4:13. 10.3389/fnsys.2010.00013. eCollection 2010. 20577591 PMC2889691

[19] Fox MD Zhang D Snyder AZ Raichle ME The global signal and observed anticorrelated resting state brain networks. J Neurophysiol (2009) 101:3270–83. 10.1152/jn.90777.2008 PMC2694109 19339462

[20] Murphy K Birn RM Handwerker DA Jones TB Bandettini PA The impact of global signal regression on resting state correlations: are anti-correlated networks introduced? Neuroimage (2009) 44:893–905. 10.1016/j.neuroimage.2008.09.036 18976716 PMC2750906

[21] Van Dijk KR Sabuncu MR Buckner RL The influence of head motion on intrinsic functional connectivity MRI. Neuroimage (2012) 59:431–8. 10.1016/j.neuroimage.2011.07.044 PMC3683830 21810475

[22] Power JD Barnes KA Snyder AZ Schlaggar BL Petersen SE Spurious but sys­tematic correlations in functional connectivity MRI networks arise from subject motion. Neuroimage (2012) 59:2142–54. 10.1016/j.neuroimage.2011.10.018 PMC3254728 22019881

[23] Tzourio-Mazoyer N Landeau B Papathanassiou D Crivello F Etard O Delcroix N Automated anatomical labeling of activations in SPM using a macroscopic anatomical parcellation of the MNI MRI single-subject brain. Neuroimage (2002) 15:273–89. 10.1006/nimg.2001.0978 11771995

[24] Lai MC Lombardo MV Chakrabarti B Sadek SA Pasco G Wheelwright SJ A shift to randomness of brain oscillations in people with autism. Biol Psychiatry (2010) 68:1092–9. 10.1016/j.biopsych.2010.06.027 20728872

[25] Supekar K Uddin LQ Khouzam A Phillips J Gaillard WD Kenworthy LE Brain hyperconnectivity in children with autism and its links to social deficits. Cell Rep (2013) 5:738–47. 10.1016/j.celrep.2013.10.001 PMC3894787 24210821

[26] Di Martino A Yan CG Li Q Denio E Castillanos FX Alaerts K The autism brain imaging data exchange: towards a large-scale evaluation of the intrinsic brain architecture in autism. Mol Psychiatry (2013) 19:659–67. 10.1038/mp.2013.78 PMC4162310 23774715

[27] Anderson JS Nielsen JA Froehlich AL DuBray MB Druzgal TJ Cariello AN Functional connectivity magnetic resonance imaging classification of autism. Brain (2011) 134:3742–54. 10.1093/brain/awr263 PMC3235557 22006979

[28] Nielsen JA Zielinski BA Fletcher PT Alexander AL Lange N Bigler ED Multisite functional connectivity MRI classification of autism: ABIDE results. Front Hum Neurosci (2013) 7:599. 10.3389/fnhum.2013.00599 24093016 PMC3782703

[29] Drysdale AT Grosenick L Downar J Dunlop K Mansouri F Meng Y Resting-state connectivity biomarkers define neurophysiological subtypes of depression. Nat Med (2017) 23:28–38. 10.1038/nm.4246 27918562 PMC5624035

[30] Benjamini Y Hochberg Y Controlling the false discovery rate: a practical and powerful approach to multiple testing. J R Stat Soc Series B Methodol (1995) 57:289–300. 10.1111/j.2517-6161.1995.tb02031.x

[31] Xia M Wang J He Y BrainNet Viewer: a network visualization tool for human brain connectomics. PLoS One (2013) 8:e68910. 10.1371/journal.pone.0068910 PMC3701683 23861951

[32] Rudie JD Brown JA Beck-Pancer D Hernandez LM Dennis EL Thompson PM Altered functional and structural brain network organization in autism. Neuroimage Clin (2012) 2:79–94. 10.1016/j.nicl.2012.11.006 24179761 PMC3777708

[33] Nair A Treiber JM Shukla DK Shih P Muller RA Impaired thalamocortical connectivity in autism spectrum disorder: a study of functional and anatomical connectivity. Brain (2013) 136:1942–55. 10.1093/brain/awt079 PMC3673456 23739917

[34] Verly M Verhoeven J Zink I Mantini D Van Oudenhove L Lagae L Structural and functional underconnectivity as a negative predictor for language in autism. Hum Brain Mapp (2014) 35:3602–15. 10.1002/hbm.22424 PMC6869461 24375710

[35] Yerys BE Herrington JD Satterthwaite TD Guy L Schultz RT Bassett DS Globally weaker and topologically different: resting-state connectivity in youth with autism. Mol Autism (2017) 8:39. 10.1186/s13229-017-0156-6 28770039 PMC5530457

[36] Ray S Miller M Karalunas S Robertson C Grayson DS Cary RP Structural and functional connectivity of the human brain in autism spectrum disorders and attention-deficit/hyperactivity disorder: a rich club-organization study. Hum Brain Mapp (2014) 35:6032–48. 10.1002/hbm.22603 PMC4319550 25116862

[37] Murdaugh DL Maximo JO Kana RK Changes in intrinsic connectivity of the brain’s reading network following intervention in children with autism. Hum Brain Mapp (2015) 36:2965–79. 10.1002/hbm.22821 PMC6869516 26058572

[38] Abbott AE Nair A Keown CL Datko M Jahedi A Fishman I Patterns of atypical functional connectivity and behavioral links in autism differ between default, salience, and executive networks. Cereb Cortex (2016) 26:4034–45. 10.1093/cercor/bhv191 PMC5027998 26351318

[39] Linke AC Jao Keehn RJ Pueschel EB Fishman I Muller RA Children with ASD show links between aberrant sound processing, social symptoms, and atypical auditory interhemispheric and thalamocortical functional connectivity. Dev Cogn Neurosci (2018) 29:117–26. 10.1016/j.dcn.2017.01.007 PMC5664206 28223033

[40] Cerliani L Mennes M Thomas RM Di Martino A Thioux M Keysers C Increased functional connectivity between subcortical and cortical resting-state networks in autism spectrum disorder. JAMA Psychiatry (2015) 72:767–77. 10.1001/jamapsychiatry.2015.0101 PMC5008437 26061743

[41] Woodward ND Giraldo-Chica M Rogers B Cascio CJ Thalamocortical dysconnectivity in autism spectrum disorder: an analysis of the Autism Brain Imaging Data Exchange. Biol Psychiatry Cogn Neurosci Neuroimaging (2017) 2:76–84. 10.1016/j.bpsc.2016.09.002 28584881 PMC5455796

[42] Fu Z Tu Y Di X Du Y Sui J Biswal BB Transient increased thalamic-sensory connectivity and decreased whole-brain dynamism in autism. Neuroimage (2019):191–204 .org/10.1016/neuroimage.2018.06.003 PMC6281849 29883735

[43] Ouhaz Z Fleming H Mitchell AS Cognitive functions and neurodevelopmental disorders involving the prefrontal cortex and mediodorsal thalamus. Front Neurosci (2018) 12:33. 10.3389/fnins.2018.00033 29467603 PMC5808198

[44] Raichle ME The brain’s default mode network. Annu Rev Neurosci (2015) 38:433–47. 10.1146/annurev-neuro-071013-014030 25938726

[45] Eddy CM The junction between self and other? Temporo-parietal dysfunction in neuropsychiatry. Neuropsychologia (2016) 89:465–77. 10.1016/j.neuropsychologia.2016.07.030 27457686

[46] Guo X Duan X Long Z Chen H Wang Y Zheng J Decreased amygdala functional connectivity in adolescents with autism: a resting-state fMRI study. Psychiatry Res Neuroimaging (2016) 257:47–56. 10.1016/j.pscychresns.2016.10.005 27969061

[47] Odriozola P Dajani DR Burrows CA Gabard-Durnam LJ Goodman E Baez AC Atypical frontoamygdala functional connectivity in youth with autism. Dev Cogn Neurosci (2018). 10.1016/j.dcn.2018.12.001 PMC6570504 30581125

[48] Rausch A Zhang W Haak KV Mennes M Hermans EJ van Oort E Altered functional connectivity of the amygdaloid input nuclei in adolescents and young adults with autism spectrum disorder: a resting state fMRI study. Mol Autism (2016) 7:13. 10.1186/s13229-015-0060-x 26823966 PMC4730628

[49] Rausch A Zhang W Beckmann CF Buitelaar JK Groen WB Haak KV Connectivity-based parcellation of the amygdala predicts social skills in adolescents with autism spectrum disorder. J Autism Dev Disord (2018) 48:572–82. 10.1007/s10803-017-3370-3 PMC5807492 29119520

[50] Shen MD Li DD Keown CL Lee A Johnson RT Angkustsiri K Functional connectivity of the amygdala is disrupted in preschool-aged children with autism spectrum disorder. J Am Acad Child Adolesc Psychiatry (2016) 55:817–24. 10.1016/j.jaac.2016.05.020 PMC5003422 27566123

[51] Herting MM Sowell ER Puberty and structural brain development in humans. Front Neuroendocrinol (2017) 44:122–37. 10.1016/j.yfrne.2016.12.003 PMC5612369 28007528

[52] Vijayakumar N de Macks Z Shirtcliff EA Pfeifer JH Puberty and the human brain: insights into adolescent development. Neurosci Biobehav Rev (2018) 92:417–36. 10.1016/j.neubiorev.2018.06.004 PMC6234123 29972766

[53] Krauth A Blanc R Poveda A Jeanmonod D Morel A Szekely G A mean three-dimensional atlas of the human thalamus: generation from multiple histological data. Neuroimage (2010) 49:2053–62. 10.1016/j.neuroimage.2009.10.042 19853042

