# Supplementary material for: Thalamocortical Hyperconnectivity and Amygdala-Cortical Hypoconnectivity in Male Patients With Autism Spectrum Disorder
Source: Front Psychiatry. 2019 Apr 16;10:252. doi: 10.3389/fpsyt.2019.00252 (PMC6482335; doi:10.3389/fpsyt.2019.00252)
Supplement: Supplementary Table 1 — Participants demographics. [file DataSheet_1.doc]

Supplementary Table 1: Participants demographics

| Institute | ASD (n) | CTL (n) | Mean age (SD) | Non rt-hander (%) | Mean IQ (ASD/CTL) |
| --- | --- | --- | --- | --- | --- |
| BNI | 13 | 9 | 20.8 (2.3) | 0 | 104/110 |
| EMC | 14 | 14 | 8.3 (1.0) | 21.4 | 100/97 |
| ETH | 7 | 20 | 22.8 (4.3) | 0 | 110/116 |
| GU | 31 | 24 | 11.1 (1.5) | 16.3 | 118/120 |
| IU | 15 | 12 | 21.5 (2.9) | 18.5 | 115/115 |
| KUL | 24 | 0 | 22 (2.9) | 16.6 | 106/- |
| KKI | 31 | 78 | 10.5 (1.3) | 15.5 | 100/114 |
| NYU | 41 | 28 | 9.4 (3.7) | 15.2 | 102/116 |
| OILH | 14 | 15 | 22.4 (3.6) | 20.6 | 112/114 |
| OHSU | 28 | 23 | 11.2 (2.1) | 1.9 | 106/119 |
| TCD | 13 | 18 | 15.4 (3.2) | 0 | 115/120 |
| SDSU | 24 | 23 | 13.1 (3.1) | 19.5 | 99/101 |
| SU | 17 | 18 | 11.0 (1.2) | 5.7 | 110/113 |
| UCD | 13 | 10 | 14.8 (1.9) | 4.3 | 104/112 |
| UCLA | 12 | 10 | 11.1 (2.3) | 13.6 | 100/113 |
| MIA | 7 | 6 | 10.7 (1.9) | 0 | 99/111 |
| USM | 7 | 7 | 19.9 (4.8) | 7.6 | 105/118 |

For abbreviation of institute see ABIDE website.

Supplementary Table 2: Names of institutes and data acquisition parameters

| Institute | Vendor | Scanner | Headcoil | B0 (T) | TR (ms) | TE (ms) | FA (deg) | Voxel Size (mm) | Slices | Volumes | Time (m) | Eyes | Note |
| --- | --- | --- | --- | --- | --- | --- | --- | --- | --- | --- | --- | --- | --- |
| BNI | Philips | Ingenia | 15Ch | 3 | 3000 | 25 | 80 | 3.75x3.75x4.0 | 50 | 120 | 6:09 | close |  |
| EMC | GE | MR750 | 8Ch | 3 | 2000 | 30 | 85 | 3.59x3.59x4.0 | 37 | 160 | 5:20 | close |  |
| ETH | Philips | Achieva | 32Ch | 3 | 2000 | 25 | 90 | 3.0x3.1x3.0 | 40 | 210 | 7:06 | open |  |
| GU | Siemens | TrioTim | 12Ch | 3 | 2000 | 30 | 90 | 3.0x3.0x3.0 | 43 | 152 | 5:04 | open |  |
| IU | Siemens | TrioTim | 32Ch | 3 | 813 | 28 | 60 | 3.4x3.4x3.4 | 42 | 433 | 16:21 | open | M.B. |
| KUL | Philips | Achieva | 32Ch | 3 | 2500 | 30 | 90 | 1.56x1.56x2.7 | 45 | 162 | 7:00 | open | M.B. |
| KKI | Philips | Achieva | 8/32Ch | 3 | 2500 | 30 | 75 | 3.0x3.0x3.0 | 47 | 128 | 5:20 | open |  |
| NYU | Siemens | Allegra | 8Ch | 3 | 2000 | 30 | 82 | 3.0x3.0x4.0 | 33 | 180 | 6:00 | open |  |
| OILH | Siemens | Skyra | 32Ch | 3 | 475 | 30 | 60 | 3.0x3.0x3.0 | 48 | 947 | 7:37 | open | M.B. |
| OHSU | Siemens | TrioTim | 12Ch | 3 | 2500 | 30 | 90 | 3.8x3.8x3.8 | 36 | 120 | 5:07 | open |  |
| TCD | Philips | Achieva | 8Ch | 3 | 2000 | 27 | 90 | 3.0x3.0x3.5 | 37 | 210 | 7:06 | open |  |
| SDSU | GE | MR750 | 8Ch | 3 | 2000 | 30 | 90 | 3.43x3.43x3.4 | 42 | 180 | 6:10 | open |  |
| SU | GE | Signa | n.a. | 3 | 2000 | 30 | 80 | 3.43x3.43x3.5 | 31 | 180 | 6:00 | close |  |
| UCD | Siemens | TrioTim | 32Ch | 3 | 2000 | 24 | 90 | 3.5x3.5x4.0 | 36 | 151 | 5:02 | open |  |
| UCLA | Siemens | TrioTim | 12Ch | 3 | 3000 | 28 | 90 | 3.0x3.0x4.0 | 34 | 120 | 6:06 | open |  |
| MIA | GE | MR750 | 32Ch | 3 | 2000 | 30 | 75 | 3.43x3.43x3.4 | 42 | 295 | 9:50 | close |  |
| USM | Siemens | TrioTim | 12Ch | 3 | 2000 | 28 | 90 | 3.4x3.4x3.3 | 40 | 240 | 8:06 | open | M.B. |
| B0; Strength of magnetic field, FA; Flip angle, Time: Scan time, Eyes; eyes were open/closed during the scan, M.B.; multiband acquisition | | | | | | | | | | | |  |  |
| Scan volumes are from representative samples. | | | | | | |  |  |  |  |  |  |  |

Supplementary Table 3: Subcortical connectivity which did not survive FDR correction.

Thalamus ***Frontal_Sup_Orb_L, Frontal_Inf_Oper_L, Rolandic_Oper_R, Rectus_L, Cingulum_Mid_L, Cingulum_Mid_R, Fusiform_R, Parietal_Sup_R, SupraMarginal_R, Precuneus_L, Paracentral_Lobule_R, Putamen_L, Pallidum_L, Temporal_Sup_L, Temporal_Sup_R, Temporal_Mid_R, Temporal_Inf_L, Temporal_Inf_R***

Amygdala Precentral_R, Frontal_Mid_R, Rolandic_Oper_L, Supp_Motor_Area_R, Insula_L, Insula_R, Hippocampus_R, ParaHippocampal_L, ParaHippocampal_R, Calcarine_R, Cuneus_L, Cuneus_R, Lingual_R, Occipital_Sup_L, Occipital_Mid_L, Occipital_Inf_L, Fusiform_R, SupraMarginal_R, Precuneus_L, Precuneus_R, Putamen_R, Pallidum_R, Temporal_Sup_L, Temporal_Mid_R

Hippocampus Frontal_Inf_Oper_R, Frontal_Inf_Tri_R, Supp_Motor_Area_R, Insula_R, Cingulum_Ant_L, Cingulum_Ant_R, Amygdala_R, Occipital_Inf_L, Occipital_Inf_R, SupraMarginal_R, Caudate_L, Caudate_R, Putamen_R, Pallidum_R, Heschl_R, Temporal_Sup_L, Temporal_Sup_R

Caudate ***Frontal_Sup_Orb_R***, ***Frontal_Inf_Orb_L***, ***Frontal_Inf_Orb_R***, ***Frontal_Med_Orb_R***, ***Rectus_L***, ***Rectus_R***, Hippocampus_L, Hippocampus_R,

Putamen Frontal_Inf_Oper_R, Frontal_Inf_Tri_R, Rolandic_Oper_L, ***Cingulum_Post_L***, ***Cingulum_Post_R***, Hippocampus_L, ParaHippocampal_L, Amygdala_L, Occipital_Inf_L, Fusiform_L, Pallidum_R, ***Thalamus_L***

Pallidum ***Cingulum_Post_R***, Amygdala_L

These connectivities are significant at p < 0.05 (uncorrected), but did not survive FDR correction at p < 0.05

Hyperconnectivity in ASD is shown in bold and italic font and hypoconnectivity in ASD is shown in plan font.

Supplementary Table 4: Correlation between FD, age, full-IQ, and mean connectivity.

| **r** | **Age** | **FD** | **FIQ** | **Conn** |
| --- | --- | --- | --- | --- |
| **Age** | **‒** | **-0.10** | **0.06** | **0.02** |
| **FD** | **-0.17** | **‒** | **-0.13** | **0.26** |
| **FIQ** | **0.06** | **-0.01** | **‒** | **0.04** |
| **Conn** | **-0.07** | **0.27** | **0.07** | **‒** |
|  |  |  |  |  |
| **p** | **Age** | **FD** | **FIQ** | **Conn** |
| **Age** | **‒** | **0.08** | **0.31** | **0.75** |
| **FD** | **0.003** | **‒** | **0.02** | **<0.001** |
| **FIQ** | **0.32** | **0.91** | **‒** | **0.48** |
| **Conn** | **0.26** | **<0.001** | **0.2** | **‒** |

Top; Pearson’s correlation coefficients between the four metrics. The results of ASD (blue, lower triangle) and CTL (orange, upper triangle) are shown. Bottom; P-values of ASD (blue, lower triangle) and CTL (orange, upper triangle) are shown. Dark color indicates significant (*p* < 0.05) results. FD: framewise displacement, FIQ: full-scale IQ, Conn: overall mean connectivity

Supplementary Table 5: Medication effect on head motion parameters

ASD on-med ASD off-med U-test

FD 0.20 (0.11) 0.23 (0.23) p=0.30

Scrubbed (%) 17.4 (14.6) 19.9 (18.9) p=0.76

FD; frame wise displacement

Scrubbed (%); the proportion of the scrubbed volumes in total scan volumes

Mean and SD in the parentheses

U-test; Mann-Whitney U-test
