# Supplementary figures and images for: Thalamocortical Hyperconnectivity and Amygdala-Cortical Hypoconnectivity in Male Patients With Autism Spectrum Disorder
Source: Front Psychiatry. 2019 Apr 16;10:252. doi: 10.3389/fpsyt.2019.00252 (PMC6482335; doi:10.3389/fpsyt.2019.00252)

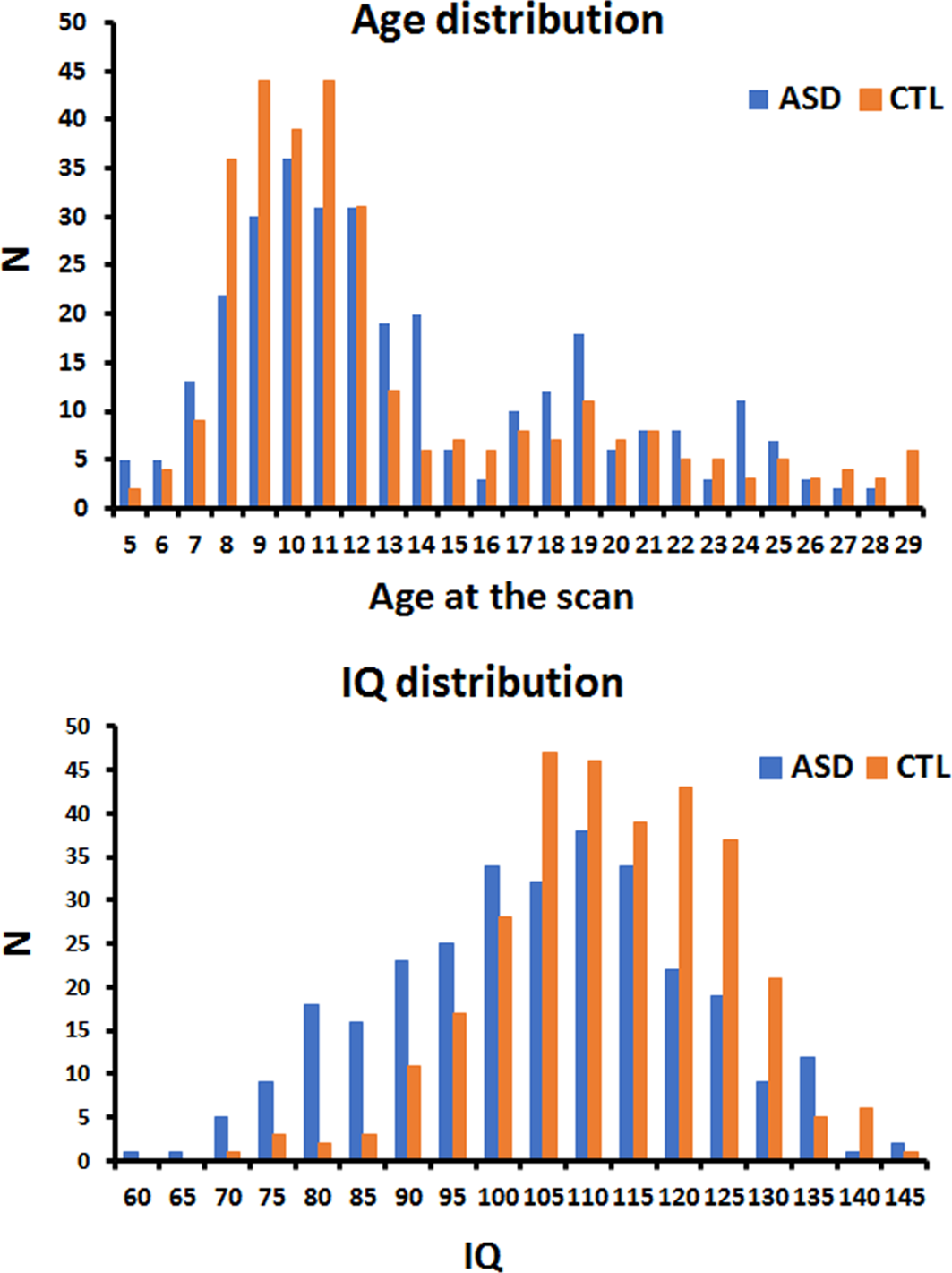

Supplement: Supplementary Figure 1 — Age and IQ distribution of the participants. Distributions of age at the time of scanning (top) and IQ (bottom) for the ASD (blue column) and CTL groups (orange column). The vertical axis indicates the number of participants. There was no significant group difference in mean age, although mean IQ was significantly lower (p< 0.001) in the ASD group than in the CTL group. IQ: intelligence quotient; ASD: autism spectrum disorder; CTL: control. [file Image_1.tif]

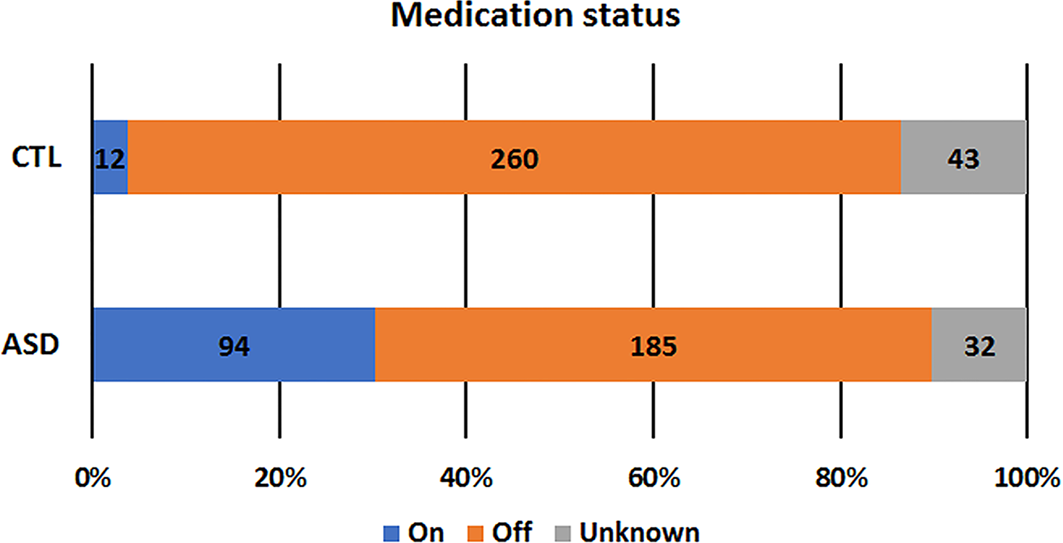

Supplement: Supplementary Figure 2 — Medication status of participants in each group. The number in the column indicates the number of participants, while the scale below indicates the percentage. [file Image_2.tif]

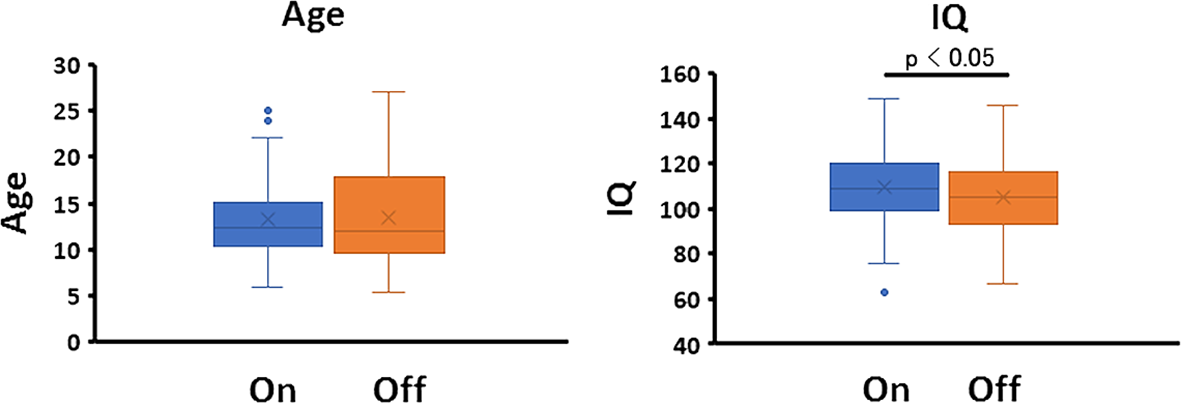

Supplement: Supplementary Figure 3 — Medication effect on age and IQ. Boxplot of age (left) and IQ (right) in the on- and off-medication ASD groups. Patients in the on-med group exhibited significantly higher mean IQ values than those of the off-med group (p< 0.05). IQ: intelligence quotient. [file Image_3.tif]
